# Unravelling the role of immune cells and FN1 in the recurrence and therapeutic process of skull base chordoma

**DOI:** 10.1002/ctm2.1429

**Published:** 2023-10-02

**Authors:** Xulei Huo, Sihan Ma, Can Wang, Lairong Song, Bohan Yao, Sipeng Zhu, Peiran Li, Liang Wang, Zhen Wu, Ke Wang

**Affiliations:** ^1^ Department of Neurosurgery Beijing Tiantan Hospital Capital Medical University Beijing China; ^2^ Department of Neuro‐oncology Cancer Center Beijing Tiantan Hospital Capital Medical University Beijing China

**Keywords:** chordoma, FN1, immunosuppressive, single cell

## Abstract

**Background:**

Skull base chordoma is a rare and aggressive tumour of the bone that has a high likelihood of recurrence. The fundamental differences in single cells between primary and recurrent lesions remain poorly understood, impeding development of effective treatment approaches.

**Methods:**

To obtain an understanding of the differences in single cells between primary and recurrent chordomas, we performed single‐cell RNA sequencing and T‐cell/B‐cell receptor (BCR) sequencing. This allowed us to delineate the differences between the two types of tumour cells, tumour‐infiltrating lymphocytes, myeloid cells, fibroblasts and B cells. Copy number variants (CNVs) were detected and compared between the tumour types to assess heterogeneity. Selected samples were subjected to immunohistochemistry to validate protein expression. Fluorescence in situ hybridisation experiments, Transwell assays and xenograft mouse models helped verify the role of fibronectin 1 (FN1) in chordoma.

**Results:**

Promoting natural killer (NK) cell and CD8_GZMK T‐cell function or inhibiting the transformation of CD8_GZMK T cells to CD8_ZNF683 T cells and promoting the transformation of natural killer T (NKT) cells to NK cells are promising strategies for preventing chordoma recurrence. Additionally, inhibiting the M2‐like activity of tumour‐associated macrophages (TAMs) could be an effective approach. Antigen‐presenting cancer‐associated fibroblasts (apCAFs) and dendritic cells (DCs) with high enrichment of the antigen‐presenting signature were enriched in primary chordomas. There were fewer plasma cells and BCR clonotypes in recurrent chordomas. Remarkably, FN1 was upregulated, had more CNVs, and was more highly secreted by tumours, macrophages, CD4 T cells, CD8 T cells and fibroblasts in recurrent chordoma than in primary chordoma. Finally, FN1 enhanced the invasion and proliferation of chordomas in vivo and in vitro.

**Conclusion:**

Our comprehensive picture of the microenvironment of primary and recurrent chordomas provides deep insights into the mechanisms of chordoma recurrence. FN1 is an important target for chordoma therapy.

## Graphical abstract

- Promoting NK and CD8_GZMK function or changing the transformation of CD8 T and NK cells may help preventing chordoma recurrence.
- Suppressing the function of M2‐TAMs may be an effective strategy.
- High apCAFs and DCs were enriched in primary chordomas and plasma cells and BCR clonotypes were rarer in recurrent chordomas.
- FN1 was an important target.

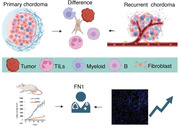

## 1 INTRODUCTION

Skull base chordoma is an uncommon and malignant tumour of the bone that arises from the leftover parts of the notochord.^1^ It can also develop in the sacrococcygeal region.^2^ Although chordoma grows slowly, especially in cases involving the skull base, it has infiltrative characteristics, resulting in frequent recurrence, malignant progression and poor progression‐free survival.^3^ Surgery and radiotherapy are the main therapeutic strategies for chordoma patients, but the clinical outcome worsens with recurrence.^4^ The recurrence of chordoma has been linked to immunodeficiency, which has been recognised as a significant contributing factor.^5^, ^6^ Earlier investigations using single‐cell RNA sequencing (scRNA‐seq) have provided insights into the interactions between tumours and the immune system, uncovered a detailed network of ligands and receptors within chordomas and identified p‐epithelial–mesenchymal transition (EMT) as a crucial molecular target for chordoma treatment.^7^, ^8^ scRNA‐seq has offered an unparalleled understanding of intratumoural transcriptomic diversity in various cancers, highlighting the significance of specific cell populations in drug resistance and tumour prognosis.^9^, ^10^ However, there is still an incomplete understanding of the heterogeneity between primary and recurrent chordoma and the mechanisms underlying chordoma recurrence.

The objective of this study was to elucidate the transcriptomic and immune landscapes of primary and recurrent chordomas using scRNA‐seq. We conducted scRNA‐seq analysis on four primary chordomas and four recurrent chordomas to comprehensively examine the characteristics of chordomas and the changes occurring in the process of recurrence. The analysis involved a total of 14 626 transcriptomes from individual cells. Prominent characteristics of EMT were observed in a distinct group of tumour‐specific cells found in recurrent chordomas. These cells displayed characteristics associated with increased invasiveness and metastatic potential. Further supporting previous findings,^11^ we observed differences in immune cell and fibroblast compositions between primary and recurrent chordomas. Recurrent chordomas were characterised by a microenvironment enriched with M2‐like tumour‐associated macrophages (TAMs) exhibiting enhanced protumour capabilities. T‐cell exclusion and reduced T‐cell cytotoxicity were also observed in recurrent chordomas, accompanied by decreased T‐cell receptor (TCR) clonality. Primary chordomas, on the other hand, were enriched with dendritic cells (DCs) and antigen‐presenting cancer‐associated fibroblasts (apCAFs) with a strong antigen‐presenting signature, while plasma cells and B‐cell receptor (BCR) clonotypes were less abundant in recurrent chordomas. EMT‐like cancer‐associated fibroblasts (eCAFs) had a strong EMT signature in recurrent chordomas, and cancer‐associated fibroblasts (CAFs) expressing fibronectin 1 (FN1) displayed elevated levels of ligands associated with EMT. Notably, FN1 was increased in tumour cells, CD4 T cells, CD8 T cells, macrophages and CAFs in recurrent chordomas. The invasion and proliferation of chordoma cells were increased by FN1, and recurrent chordomas showed a higher rate of copy number gain. These findings highlight the therapeutic potential of targeting FN1 as a critical factor in chordoma recurrence. Overall, our research offers an important understanding of the diversity at the molecular and cellular levels of primary and recurrent chordomas, illuminating possible targets for therapy and approaches to treat this challenging bone tumour.

## 2 METHODS

### 2.1 Chordoma samples

Chordoma samples utilised for sequencing in this research were acquired from eight individuals who received surgical intervention at Beijing Tiantan Hospital, Capital Medical University, between January and December 2022. According to the 2021 WHO classification of bone tumours, all individuals were diagnosed with classical chordoma through pathological examination. Immunohistochemistry (IHC) confirmed strong positive expression of brachyury T (TBXT)) in all chordoma cells (Figure S1). Fresh tumour samples were collected during surgery, and written informed consent was acquired from every individual. Table S1 contains further information about the patients and their samples.

### 2.2 cRNA sequencing

After sampling brain tumour tissue during surgery, the tissue was washed with physiological saline to remove blood stains. The tissue was placed into a brown sample storage tube containing MACS Tissue Storage Solution (Miltenyi Biotec). The tube was transported to the laboratory at 4°C, and the tissue was dissociated within 1 h. The tissue was retrieved from the sample storage tube and placed into cold phosphate‐buffered saline (PBS) for washing. Afterward, the specimen was sliced into 2–3‐mm fragments and transformed into a solitary‐cell mixture utilising the Miltenyi Biotec Brain Tumour Dissociation kit. The Red Blood Cell Lysis Solution from Miltenyi Biotec was utilised for the lysis of red blood cells, while the Dead Cell Removal Kit, also from Miltenyi Biotec, was employed to eradicate deceased cells. The cells were suspended in PBS with .04% bovine serum albumin (BSA) and RNA inhibitor (1 U/μL). The LUNA‐FL Automated Fluorescence Cell Counter machine was used for cell staining with AcridineOrange/Propidiumiodidep (AO/PI) dye to assess cell viability, concentration, aggregation rate and other parameters. To produce Gel Bead in emulsions (GEMs) capturing 10 000 cells per sample, the 10× Genomics Chromium Controller device was employed in conjunction with the Chromium Next GEM Single Cell 5′ Reagent Kits v2 (Dual Index). After amplifying the cDNA, single‐cell 5′ transcriptome libraries were constructed using 50 ng of the cDNA. The Chromium Single Cell Human TCR Amplification Kit was utilised for TCR sequencing, with 2 μL of cDNA. Likewise, the Chromium Single Cell Human BCR Amplification Kit was employed for BCR sequencing. The Library Construction Kit was utilised to construct TCR and BCR libraries. To evaluate the libraries' quality and concentration on the Qubit 4.0 instrument, the Qubit 1× dsDNA Assay Kit was employed. The StepOnePlus Real‐Time PCR System was employed to ascertain the molar concentration of the libraries. The LabChip Touch system was employed to evaluate the insert size of the libraries. Finally, the Illumina NovaSeq6000 sequencing platform was used for paired‐end 150 base‐pair sequencing.

### 2.3 Data processing

Cell Ranger (version 6.0.1) was used to process the original fastq files obtained from sequencing, utilising default mapping parameters and the *Homo sapiens* GRCh38 genome (reference). Further analysis excluded cells that had a significant proportion (>20%) of transcript counts originating from genes encoded by mitochondria. The R package DoubletFinder was utilised to detect and eliminate potential doublets, assuming a doublet rate of 5%.^12^ For downstream analysis, cells that had over 200 distinct molecular identifiers (UMI) were accounted. The clustering analysis was conducted on a per‐sample basis using Seurat.^13^ Qualified cells were chosen to create the processed digital gene expression (DGE) matrix, which included genes expressed in a minimum of three cells. After applying the natural logarithm to the DGE matrix by using the equation ln (counts per million (CPM)/100 + 1), the gene count was eliminated through regression. For the initial principal component (PC) analysis, approximately 2000 genes were chosen that had an average expression above .01 and a dispersion exceeding .45.

The PCElbowPlot function and JackStrawPlot function were used to determine the number of PCs for the following uniform manifold approximation and projection (UMAP) analysis. To achieve a more compact arrangement, UMAP was utilised for visualisation, utilising a minimum distance (min.dist) value of .25. The clustering analysis in the ‘FindAllCluster’ function utilised various resolution parameters, which ranged from .1 to 1.0. The resolution and quantity of personal computers were modified for each individual sample. By analysing the variation in gene expression between clusters, we ultimately established distinct cluster numbers. The FindAllMarkers function in Seurat was used to perform the default Wilcoxon rank‐sum test to identify differentially expressed markers in each cluster. The analysis utilised the following parameters: min.pct = .25, min.diff.pct = .25 and logfc.threshold = .25. An extensive review of the literature and searching for specific gene expression patterns were used to annotate cell types.

### 2.4 Functional enrichment analysis

After annotating each type of cell, we performed Gene Ontology (GO) functional enrichment analysis using the clusterProfiler package (version 4.6.2) and the org.Hs.eg.db package (version 3.16.0)^11^ to detect genes that were differently expressed among various clusters. The objective of this analysis was to clarify the biological mechanisms and possible roles linked to various cell categories. For the GO analysis, a significance level of .05 was used as the *p*‐value threshold. Furthermore, we utilised gene set enrichment analysis (GSEA) using curated gene sets to detect pathways that were either stimulated or suppressed among the cell clusters. A modified version of the competitive gene set enrichment test called CAMERA, developed by Cillo et al., was utilised to perform the GSEA.^14^ The analysis was conducted using the R package SingleSeqGset (version 0.1.2). To summarise, the average gene expression level was computed, and the natural logarithm of the difference in expression (fold change (FC)) between a particular cell cluster and the remaining cells was employed as the statistical measure for analysis.^14^ We employed the 50 hallmark gene sets from the MSigDB databases (https://www.gsea‐msigdb.org/gsea/msigdb)^15^ for the GSEA.

### 2.5 Pathway activity score

To evaluate the enrichment of pathways using the most highly expressed genes in each individual cell, we utilised a technique known as AUCell.^7^, ^16^ To determine the activity score for each pathway, this approach required calculating the proportion of expressed genes in the query gene set and their relative expression rank compared to other genes within each cell. To evaluate the EMT score in tumours and CAFs, we obtained the EMT‐related gene list from MSigDB.^17^ This gene list was used to evaluate the EMT score in tumour cells and eCAFs. To calculate the signature score of antigen presentation in DCs and apCAFs, we obtained the gene list from the REACTOME_MHC_CLASS_II_ANTIGEN_PRESENTATION pathway (c2.cp.reactome.v7.2.symbols.gmt) through the MSigDB website.^18^ To investigate the cytotoxic and exhausted functions of tumour‐infiltrating lymphocytes (TILs), we computed the cytotoxic and exhausted scores for individual cells by utilising established markers. The score for cytotoxicity was calculated based on genes related to cytotoxicity (GZMA, GZMB, GZMK, GNLY, IFNG, PRF1 and NKG7), whereas the score for exhaustion was determined using genes associated with exhaustion (LAG3, TIGIT, PCCD1, HAVCR2, CTLA4, LAYN and ENTPD1). To estimate the macrophage phenotype (M0, M1 or M2), we employed the gene list from the ‘LM22.xls’ file obtained from CIBERSOR.^19^

### 2.6 Single‐cell copy number variation analysis

We used the inferCNV package (version 1.15.3)^20^ to calculate the approximate number of initial copy number variants (CNVs) present in the tumour cells and immune cells were used as the reference. First, we excluded cells that had less than 2000 UMIs. Next, the analysis was conducted using the default settings of the hidden Markov model (HMM) and a denoising cutoff of .1. Based on the CNV values generated by the HMM, the ‘subcluster’ method was applied to infer subcluster cells. We utilised genomic cytoband data to annotate the CNV alterations at the arm level and transformed p‐ or q‐arm‐level changes into corresponding CNVs according to their positions. Every CNV was classified as either an increase or a decrease. After converting and annotating the data, subclones that had the same arm‐level CNVs were combined, and the trees were rearranged to depict the CNV structure of the subclones. We employed the UPhyloplot2 algorithm, created by Durante et al., to visually represent the data,^21^ which can be found at https://github.com/harbourlab/UPhyloplot2. This algorithm automated the generation of intratumour evolutionary trees. The visualisation included a scalable vector graphics file for each sample, which represented the phylogenetic tree and could be scaled. Each branch in the tree had a length that was directly related to the number of cells it represented, and it also included a spacer (circle diameter + 5 pixels).^21^ The inferCNV HMM subcluster CNV prediction method generated CNV calls at the arm level, and the input for visualisation was the proportion of cells in each subclone.

### 2.7 RNA velocity analysis

Once the BAM files were acquired from the Cell Ranger Analysis pipeline, they were brought into the Velocyto pipeline for the purpose of producing loom files that encompass count matrices for both spliced and unspliced reads. The scVelo package (version 0.2.4)^22^ was employed to compute gene‐specific velocities, which indicate the steady‐state dynamics of gene expression within cells, using the aforementioned loom files. The cell transition status based on these velocities was visualised in the original UMAP embedding, providing insights into the cellular trajectory and state transitions.

### 2.8 Pseudotime trajectory analysis

To examine the developmental progression of TILs and myeloid cells, trajectory analysis was conducted using the monocle package (version 2.28.0).^23^ Each group was analysed with specific parameters, including a lower detection limit of .5, minimum expression of .1 and a requirement of at least 10 cells expressing the genes of interest. The function plot_cell_trajectory was used to display the potential path using pseudotime, Seurat clusters and the provided data.

### 2.9 Single‐cell V(D)J analysis

Default settings were used to process the TCR/BCR sequencing data using the CellRanger V(D)J pipeline (version 2.0.0) provided by 10× Genomics. The information was aligned with the human VDJ reference genome GRCh38. Afterward, clonotype analysis was conducted utilising scRepertoire software (version 1.6.1).^24^ The steps for processing the data adhered to the instructions outlined in the scRepertoire GitHub repository (https//github.com/ncborcherding/scRepertoire). The dataset was used to classify clonotypes into various states according to their frequency. The specified clonotype categories were hyperexpanded (50 < *X* ≤ 150), large (20 < *X* ≤ 50), medium (5 < *X* ≤ 20), small (1 < *X* ≤ 5) and single (0 < *X* ≤ 1), where *X* denotes the quantity of cells containing the clonotype.

### 2.10 Fluorescence in situ hybridisation experiments

To assess and identify the presence and distribution of FN1 in chordoma tissues, fluorescence in situ hybridisation (FISH) tests were performed. In short, following prehybridisation at a temperature of 37°C for a duration of 1 h, the sections were subjected to hybridisation with FN1 probes labelled with Cy3 (General Biol) at a temperature of 40°C overnight and subsequently stained with 4`,6‐diamidino‐2‐phenylindole (DAPI). Photographs of the slides were taken using a fluorescence microscope from Leica.

### 2.11 Cellular communications

For the examination of interactions between cells, we utilised CellChat software (version 1.6.1).^25^ The examination relied on an extensive collection of recognised ligands, receptors and their associations. To infer intercellular communications, the following steps were taken: (1) recognising signalling genes with differential expression, (2) computing an average expression ensemble, and (3) estimating the probabilities of intercellular communication. By applying a permutation test, we determined which ligand‒receptor interactions were enriched in the identified cell subsets. Ligand‒receptor pairs that had a *p*‐value less than .05 were deemed to be statistically significant and were subsequently examined for their possible functional roles in facilitating cell‐to‐cell communication.

### 2.12 IHC staining and multiplex IHC

For IHC, the samples were sectioned into 5 μm slices. The tissue samples were warmed in an oven at a temperature of 60°C for a duration of 1 h and then underwent deparaffinisation and rehydration using varying levels of alcohol concentrations as per established procedures. To obtain antigens, the sections that had been deparaffinised were placed in a microwave oven and heated at low temperature for 25 min after being immersed in a solution for antigen retrieval (Table S2). The activity of endogenous peroxidase was inhibited by treating the sections with a blocking solution for 10 min. To prevent nonspecific immunoglobulin binding, the sections were incubated with normal goat serum for 1 h. The slides were subsequently treated with a primary antibody (Table S2), either overnight at 4°C or for 2 h at room temperature. Following incubation, the slides were treated with an IHC enhancer for a duration of 20 min to amplify the staining signal. Afterward, incubation took place with a related secondary antibody that was linked to horseradish peroxidase at 37°C for 1 h. To develop the colour reaction, 3,3′‐diaminodbenzidine substrate was introduced, followed by a 5‐min counterstaining with haematoxylin on the sections. Finally, the samples were dried, cleared and placed in a watery mounting substance for examination under a microscope.

In the case of multiplex IHC (mIHC), the procedure involved the same steps until incubation with primary antibodies. Following incubation with the primary antibody, the samples were treated with secondary antibodies labelled with fluorescence from the Opal 7‐Colour Manual IHC Kit (PerkinElmer) for a duration of 2 h at ambient temperature. Afterward, the slides were exposed to DAPI solution for 5 min in the absence of light to stain the nuclei of the cells. To eliminate any unbound antibodies or staining reagents, the tissues were rinsed in metal‐free PBS for 10 min. To protect the stained tissues, the slides were mounted with Vectashield Plus Antifade Mounting Medium (Vector Laboratories) using coverslips. Finally, to secure the coverslips in place, clear nail polish (Revlon 771) was applied as a sealant.

### 2.13 Semiquantitative analysis of IHC

To ensure reliable and consistent evaluation of the IHC results, a rigorous assessment process was implemented. The samples were reviewed by two pathologists who were skilled in assessing IHC and worked independently. Crucially, the clinical outcome of the patients was concealed from them, thereby reducing possible bias. The evaluation of protein marker expression involved assessing two important factors: the proportion of cells showing positive staining and the intensity of the staining. The proportion of cells showing immune reactivity was assessed as 0 points (less than 10% of cells being positive), 1 point (10%–50% of cells being positive), or 2 points (more than 50% of cells being positive). Staining intensity was assessed using a scale ranging from 0 to 2, where 0 represents absence or weak staining, 1 indicates moderate staining and 2 signifies strong staining. The sum of the two scores was used to determine the overall marker expression score. The combined evaluation of both parameters led to an overall score that varied between 0 and 4.

### 2.14 Chordoma cell line culture

The human chordoma cell lines U‐CH1 and UM‐chor1 were used as described.^26^ The culture medium consisted of Iscove's Modified Dulbecco's Medium (Gibco [Life Technologies, Thermo Fisher Scientific]), Roswell Park Memorial Institute 1640 (4:1) (Gibco [Life Technologies, Thermo Fisher Scientific]), 10% foetal bovine serum (FBS; Gibco [Life Technologies, Thermo Fisher Scientific]) and 1% penicillin and streptomycin (100 mg/mL; Invitrogen). The cells were cultivated in a moist incubator with an atmosphere consisting of 5% carbon dioxide and 95% air and maintained at a temperature of 37°C.

### 2.15 Lentiviral shRNA vector construction and cell infection

To suppress the manifestation of FN1, RNA interference was utilised by employing specific shRNAs that target FN1 (shFN1), in addition to a negative control (shCtrl). These shRNA sequences were designed and synthesised by Shanghai Gene Institution. For the purpose of transporting the shRNAs into the desired cells, they were connected to BR‐V‐108 lentiviral vectors, which were supplied by Shanghai Biosciences, Co., Ltd. Lentiviral vectors are widely utilised to effectively and reliably transport genetic material into cells. After constructing lentiviral vectors containing shFN1 and shCtrl, U‐CH1 and UM‐chor1 cells were infected with the lentiviruses. Western blotting was used to evaluate the expression of FN1.

### 2.16 Western blotting

Proteins were obtained using SDS cell lysis buffer (P0013G; Beyotime) containing protease inhibitor cocktail (87785; Thermo Scientific). To extract proteins, the cells were lysed with SDS cell lysis buffer (P0013G; Beyotime) containing a mixture of protease inhibitors (87785; Thermo Scientific). The protein concentration was determined using the Pierce BCA protein assay kit (PC0020; Solarbio). The protein bands were visualised by following traditional procedures for Western blotting. The presence and abundance of the target proteins were determined by detecting the bound primary antibodies using secondary antibodies.

### 2.17 Matrigel invasion experiment

To perform the Transwell invasion assay, 100 μL of Matrigel that had been melted overnight (diluted with serum‐free medium at a ratio of 1:8, cat# 354277; Corning) was added to the upper chamber of a Transwell plate (353097; Falcon). Afterward, the plate was placed in an incubator set at a temperature of 37°C for a duration of 1 h to facilitate the solidification of the Matrigel. Next, the liquid was extracted, and the upper and lower chambers were subsequently filled with 100 and 600 μL of medium without serum. To achieve equilibrium, the chamber was maintained at a temperature of 37°C for a duration of 30 min, and any surplus medium was meticulously removed. A suspension of cells with a volume of 100 μL and a concentration of 2 × 10^4^ cells per well was prepared. Next, the cell suspension was introduced into the upper compartment of the Transwell plate. Third, the lower chambers were supplemented with 600 μL of culture medium containing 10% FBS. Following a 24‐h period, any cells that were still present in the upper chamber were meticulously eliminated by gently wiping them using a cotton swab. Finally, the cells that had penetrated the lower chamber were treated with formaldehyde and stained using .1% crystal violet dye. Total cells were counted and photographed in five areas, usually seen under a 200× magnification.^27^

### 2.18 Xenograft mouse tumour model

For the in vivo experiment, we used 4‐week‐old female BALB/c nude mice obtained from Beijing Vital River Laboratory Animal Technology Co., Ltd. in China. Two groups, shFN1 and shCtrl, were formed by dividing the mice. After trypsinisation, UM‐chor1 cells were either with or without FN1 knockdown and then adjusted to a concentration of 5 × 10^7^ cells/mL. Tumour growth was observed by monitoring the injection of a cell suspension, measuring 200 μL, into the right forearm of every mouse. During the 18‐day experimental period, the size of the tumour and the weight of the mouse were measured every other day. After the trial, the mice were compassionately put to sleep, and the tumours were removed, measured and captured in photographs. TBXT, Ki‐67 and FN1 antibodies were utilised to perform IHC on the collected tumour tissues. IHC enabled the visualisation and evaluation of the expression of these indicators in the tumour tissues. The guidelines and regulations of the Institutional Animal Care and Use Committees of Beijing Tiantan Hospital were adhered to for all experimental procedures involving animals and their corresponding protocols.

### 2.19 Statistical analysis

R 4.0.2 (https://www.rproject.org/) was utilised for conducting bioinformatics analyses and constructing graphs. Student's *t*‐test was used to compare paired groups. The calculation of Pearson's correlation coefficients was performed for the continuous variables. The chi‐square test was used to analyse categorical variables, either with or without Yates's continuity correction. Cox regression was utilised to conduct both univariate and multivariate analyses. To guarantee the dependability of the findings, each experiment was conducted independently on a minimum of three occasions. The quantitative data are presented as the mean ± SD.

## 3 RESULTS

### 3.1 Distinct heterogeneity of TME between primary and recurrent samples

**FIGURE 1:**
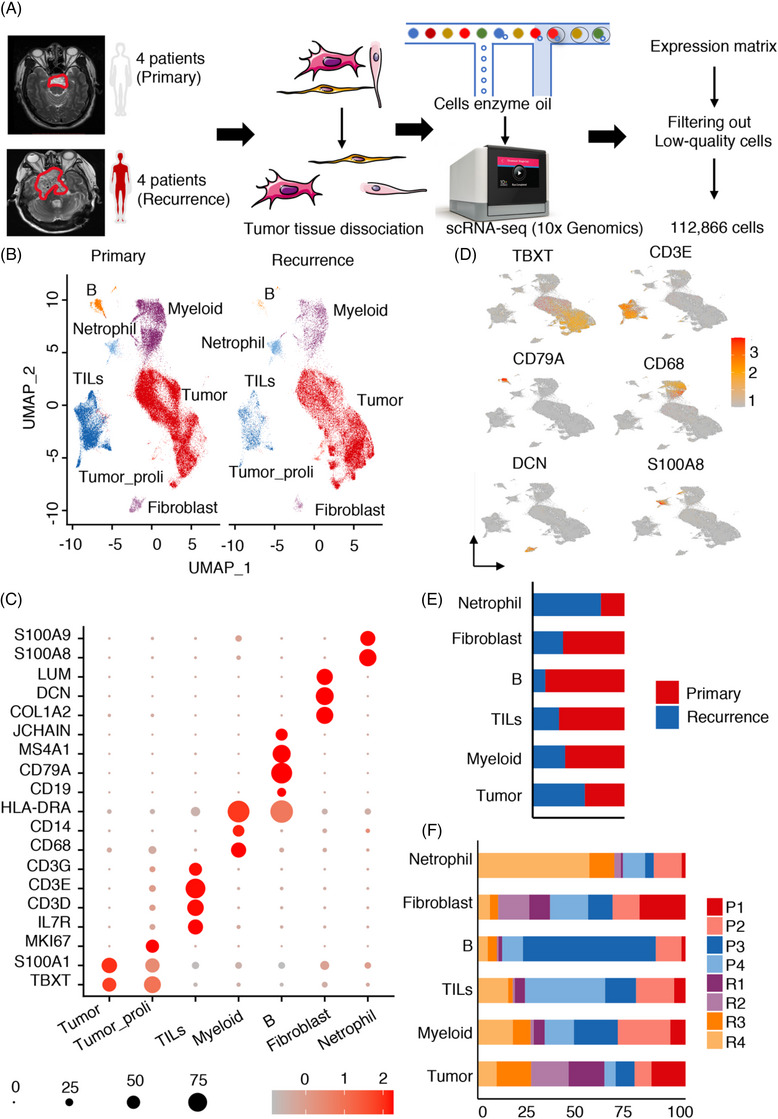
Study design and identification of cell types in primary and recurrent chordomas by single‐cell RNA sequencing (scRNA‐seq). (A) The workflow involved the collection and processing of fresh chordoma samples for scRNA‐seq. Four samples were obtained from primary chordomas, and another four samples were obtained from recurrent chordomas. (B) The major cell populations were visualised using uniform manifold approximation and projection (UMAP), with differently coloured dots representing different cell types. (C) A dot plot was drawn to display the expression of 19 signature genes across seven cellular clusters. The size of the dots indicates the proportion of cells expressing a specific marker, and the colour spectrum indicates the mean expression levels of the markers. (D) A UMAP plot was drawn to depict the expression patterns of selected genes in different cell types. (E) The fractions of each sample type relative to the total cell count per cell type are presented as sample‐type fractions. (F) The fractions of each sample type relative to the total cell count per cell type are presented as sample fractions.

To study the composition and development of the TME, we performed scRNA‐seq analysis of four recurrent tumour tissues and four primary tumour tissues obtained from chordoma patients (consisting of five males and three females, aged between 11 and 48 years). This investigation was supported by Figure 1A and Table S1. Following the implementation of quality control measures and filtering procedures, we managed to preserve a total of 112 866 cells of exceptional quality. This count encompasses 72 212 cells derived from primary lesions and 40 081 cells obtained from recurrent lesions (Figure 1A,B). Tumour samples were used to create single‐cell suspensions, which were then sequenced using the 10× Genomics Chromium system (Figure 1A, refer to Methods for further information). The presence of Brachyury, a marker protein for chordoma, was confirmed in all tumour samples through histopathological evaluation (Figure S1).^28^ Cell types were coarsely identified by merging the data and conducting various analyses, including gene expression normalisation, scaling, dimension reduction, batch correction and cell clustering. UMAP analysis identified seven primary clusters of cells, unbiasedly determined by their gene profiles and canonical markers (Figure 1C and Table S3). Figure 1C illustrates a dot plot that was created to compare the ratios of cells that exhibit distinct markers for each cluster and their scaled relative levels of expression. The clusters that were identified are as follows: (1) tumour cells that express TBXT and S100A1 at high levels^7^; (2) proliferating tumour cells highly expressing TBXT, S100A1 and MKI67; (3) TILs highly expressing IL7R, CD3D, CD3E and CD3G; (4) myeloid cells characterised by high CD14, HLA‐DRA and CD68 expression; (5) fibroblast cells specifically expressing the markers decorin (DCN), LUM and COL1A2; (6) B cells specifically expressing JCHAIN, MS4A1, CD79A and CD19; and (7) neutrophils specifically expressing S100A8 and S100A9. Chordoma cells showed the highest abundance among the cell types (Figure S2). Overall, the TME was mainly composed of chordoma cells, TILs and myeloid cells (Figure S2). Figure 1D displays the expression patterns of selected genes in distinct cell populations.

The distribution of the primary cell categories in the TME showed notable diversity among various samples, suggesting the heterogeneous composition of the chordoma TME (Figures 1E and S3). Tumour cells and neutrophils were abundant in recurrent chordomas, and other cells, including fibroblasts, TILs, B cells and myeloid cells, were abundant in primary chordomas (Figure 1E). Neutrophils were markedly depleted in primary chordomas compared with recurrent chordomas (Figure 1E), implying that tumour cells of primary lesions may escape elimination by circulating neutrophils. In each individual lesion, we noticed the presence of nearly all categories of cell populations, suggesting the existence of intertumoural heterogeneity and consistency among the lesions (Figure 1F). To summarise, our findings demonstrated significant variability in the TME between primary and recurrent samples.

### 3.2 Promoting natural killer and CD8_GZMK T‐cell function or changing the transformation of CD8 T and natural killer cells may be promising strategies for preventing chordoma recurrence

**FIGURE 2:**
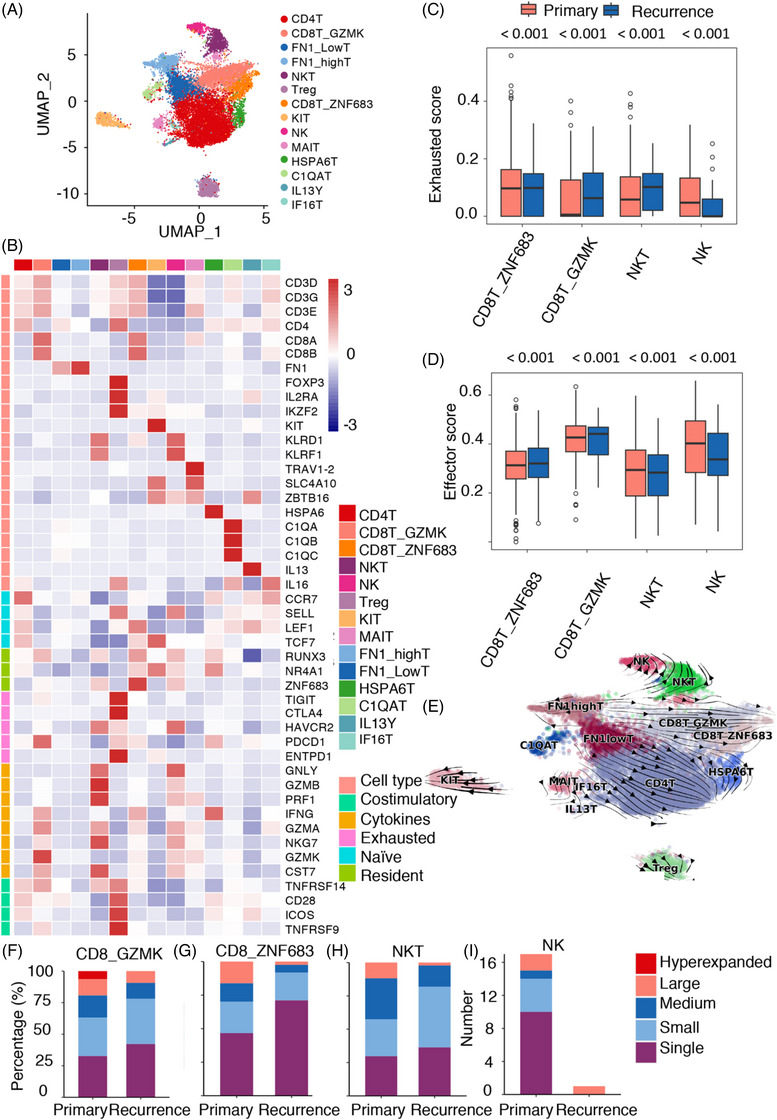
Tumour‐infiltrating lymphocyte (TIL) annotation and functional characterisation. (A) The major cell populations of TILs were visualised using uniform manifold approximation and projection (UMAP), with differently coloured dots representing different cell types. (B) Heatmap of the normalised expression of canonical TIL marker genes among clusters. (C) UMAP visualisation showing the RNA velocity of tumour cells. The distribution of the exhaustion score (D) and effector score (E) of CD8T_ZNF683 T, CD8T_GZMK T, NKT and natural killer (NK) cells between primary and recurrent chordomas. Distribution of clone type in CD8T_GZMK T (F), CD8T_ZNF683 T (G), NKT cells (H) and NK cells (I).

According to Figure 1B, TILs were highly prevalent among the cell types in the TME of chordoma. TILs have an impact on the advancement of tumours and their metastasis.^10^, ^29^ To further understand the heterogeneity of TILs in chordoma, we performed unsupervised clustering analysis on a population of 18 615 TILs. This clustering analysis identified 14 distinct subclusters of TIL cells, each characterised by unique signature genes (Figures 2A and S4 and Table S4). They are listed as follows in the format cluster number: cluster name (signature gene(s))—C0: CD4 T (CD4); C1: CD8_GZMKT (CD8A, CD8B, GZMK); C2: FN1 low T (FN1 low and ECRG4 low); C3: FN1 high T (FN1 high and ECRG4 high); C4: NKT (D3D, CD3E, CD3G, KLRF1, KLRD1); C5: Treg (FOXP3, IL2RA, IKZF2, CD4); C6: CD8_ZNF683T (CD8A, CD8B, ZNF683); C7: Mast (KIT); C8: NK (KLRF1, KLRD1); C9: MAIT (TRAV1‐2, SLC4A10, and ZBTB16); C10: HSPA6T (HSPA6); C11: C1QAT (C1QA, C1QB, C1QC); C12: IL13T (IL13); and C13: IF16T (IF16).

To determine the differences between primary and recurrent lesions, we calculated the proportion of cells in each cluster among all cells in primary and recurrent lesions and found that the proportions of CD4 T cells, CD8_GZMK T cells and CD8_ZNF683 T cells in recurrent chordomas were lower than those in primary lesions (Figure S5), indicating that there might be a more immunosuppressive state in recurrent lesions. To confirm these results, we performed IHC on 10 primary samples and 10 recurrent samples and observed an enrichment of CD4 and CD8 cells in primary chordomas. Conversely, recurrent chordomas showed an enrichment of Treg cells (Foxp3) and natural killer (NK) cells (CD56) (Figure S6).^30^ Despite the elevated proportion of Tregs observed in recurrent chordomas (Figure S5), the cells exhibited significant expression of TIGHT, CTLA4, PDCD1, ENTPD1 and HAVCR2 (Figure 2B), indicating the immunosuppressive nature of Tregs. One of the primary ways in which chordoma lesions exhibit immunosuppression is through the identification of CTLA4 and PDCD1 expression on Tregs.^31^, ^32^ Additionally, CD4 T‐cell clusters had widespread overexpression of naïve markers (CCR7, SELL, LEF1 and TCF7) (Figure 2B), suggesting that CD4 T cells were in a naïve state in chordoma.

Subsequently, our attention shifted towards CD8 T lymphocytes and NK cells due to their ability to induce cell death. Figure 2B shows that CD8_GZMKT, CD8_ZNF683T, NKT and NK cells exhibited a significant level of cytotoxicity, which was identified by the expression of GZMK, GZMB and GNLY. The examination of NKT and NK cells in chordoma revealed their elevated cytotoxicity, which was distinguished by the presence of PRF1, GZMB and GNLY. Apoptosis is induced by these molecules, resulting in the elimination of target cells. CD8_ZNF683 cells exhibited elevated levels of resident genes, including ZNF683, RUNX3 and NR4A1. Additionally, they displayed reduced levels of costimulatory molecules while showing elevated levels of CCR7 and SELL, indicating a naïve state. Conversely, CD8_GZMK cells exhibited comparatively elevated levels of costimulatory factors such as TNFRSF14, CD28, ICOS and TNFRSF9, which play a role in enhancing the cytotoxic functions of T cells. Compared to CD8_ZNF683 cells, CD8_GZMK T cells exhibited elevated levels of cytokines, including IFNG, GZMK, GZMA, NKG7 and CST7. These findings suggest that CD8_GZMK T cells represent a more mature and activated subset of CD8 T cells with enhanced cytotoxic capabilities, while CD8_ZNF683 T cells exhibit a naïve phenotype. The high expression of cytotoxic molecules and cytokines in CD8_GZMK T cells highlights their potential role in immune responses against chordoma cells. The proportion of CD8_GZMK T cells was lower in recurrent chordomas than in primary chordomas (Figure S5). In addition, the exhaustion score was high in recurrent chordomas (Figure 2C). For the CD8_ZNF683 T cells, their cytotoxic signature was decreased (Figure 2D), which may be because the cells could reside in the tumours. However, the fraction of CD8_ZNF683 T cells was decreased in recurrent chordomas (Figure S5). Tissue‐resident CD8 T cells were expanded after surgery to calculate the cytotoxic score, and the cells were remodelled into a naïve phenotype. Furthermore, trajectory analysis indicated that CD8_GZMK T cells could differentiate into CD8_ZNF683 T cells (Figures 2E and S7). The findings indicated that effector CD8 T cells undergo differentiation into naïve CD8 T cells in chordoma. Second, RNA trajectory analysis revealed that NKT cells could differentiate into NK cells (Figures 2E and S8). Intriguingly, NKT and NK cells had a higher fraction in recurrent chordomas than in primary chordomas (Figure S5). NK cells had a higher exhaustion score in primary chordomas (Figure 2C). The cytotoxic signatures for NKT and NK cells were significantly decreased in recurrent chordomas (Figure 2D). Finally, to clarify the diversity of T‐cell clonotypes in primary and recurrent samples, we conducted single‐cell sequencing on libraries enriched with V(D)J regions from the samples (see Figure S9). A greater expansion of TILs and clonotypes was observed in the primary chordoma samples than in the recurrent samples (Figure S10). Furthermore, we found that primary chordomas had a greater number of TCR clonotypes in CD8_GZMK T, CD8_ZNF683 T, NKT and NK cells (Figures 2F–I and S11). In conclusion, recurrent chordoma has fewer cytotoxic TIL proportion and TCR clonotypes. These results suggest that promoting NK and CD8_GZMK T‐cell function or inhibiting the transformation of CD8_GZMK T cells to CD8_ZNF683 T cells and promoting the transformation of NKT cells to NK cells will be promising strategies for preventing chordoma recurrence.

### 3.3 Suppressing the function of M2‐TAMs may be an effective strategy in chordoma

**FIGURE 3:**
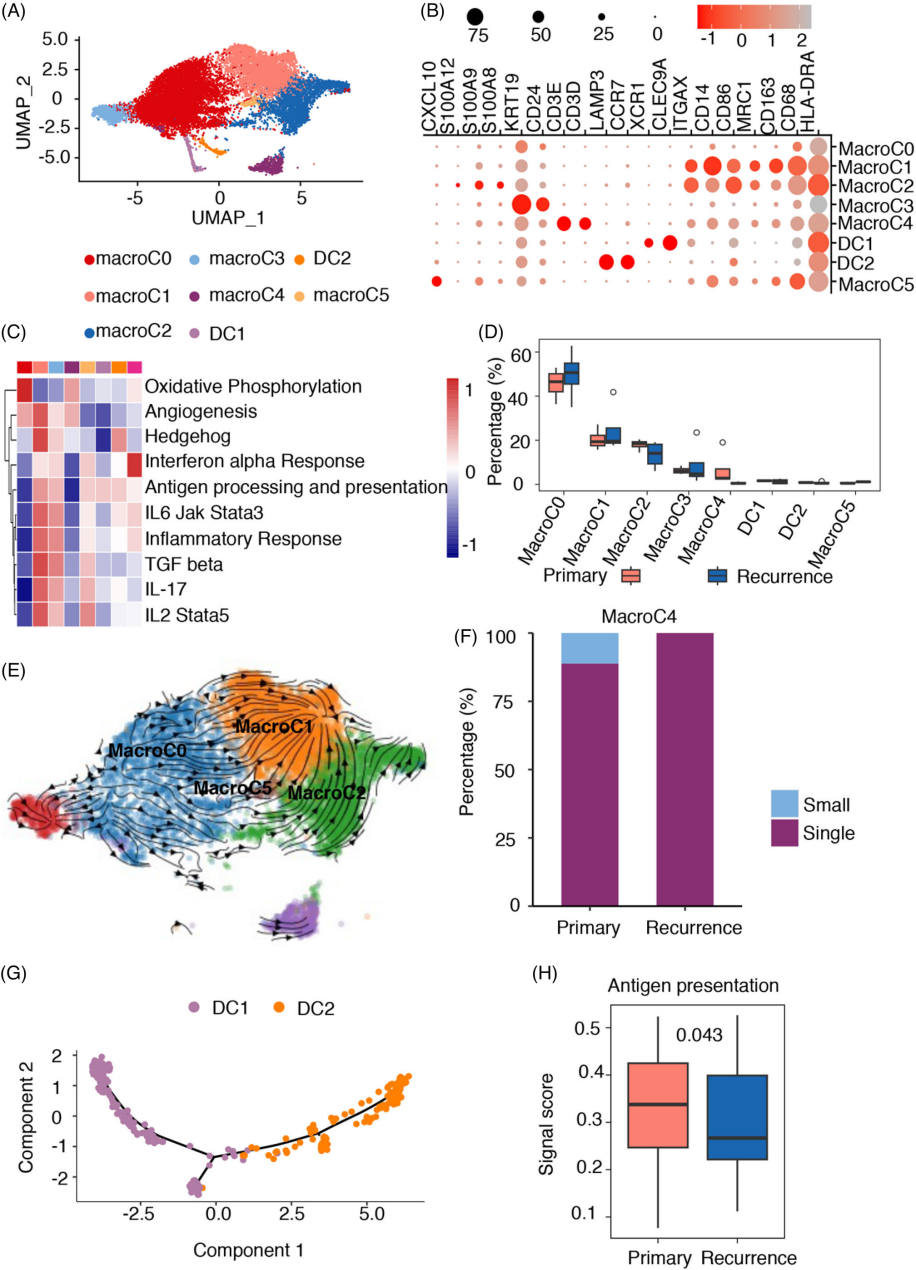
Myeloid cell annotation and functional characterisation. (A) The major cell populations of myeloid cells were visualised using uniform manifold approximation and projection (UMAP), with differently coloured dots representing different cell types. (B) Dot plots were drawn to display the expression of 19 signature genes across eight cellular clusters. The size of the dots indicates the proportion of cells expressing a specific marker, and the colour spectrum indicates the mean expression levels of the markers. (C) Gene set enrichment analysis (GSEA) results of myeloid cells. (D) Distribution of different cell types, ranked by the median frequency value. (E) UMAP visualisation showing the RNA velocity of macrophages. (F) Distribution of clone type in macroC4. (G) The Monocle 2 trajectory plot revealed the dynamic changes in dendritic cells (DCs), showcasing their developmental trajectory. (H) Boxplots comparing the average antigen presentation signature scores between DCs in primary chordoma samples and recurrent chordoma samples.

In the TME of chordoma (Figure 1B), myeloid cells, the second most prevalent cell population, make significant contributions to the progression and spread of the tumour.^33^ The study involved the analysis and clustering of 15 677 myeloid cells, which led to the discovery of eight unique subpopulations (as shown in Figure 3A,B and Table S5). Six TAM subpopulations had various features: (macroC0) CD68 CD163 low TAMs (HLA‐DRA, CD68 low, CD163 low, MRC1 low); (macroC1) CD68 CD163 high TAMs (HLA‐DRA, CD68 high, CD163 high, MRC1 high); (macroC2) monocyte‐TAMs (HLA‐DRA, CD68, CD163, MRC1, VCAN, FCN1, S100A8, S100A9, S100A12); (macroC3) KRT19 TAMs (HLA‐DRA, CD68, KRT19, CD24); (macroC4) TCR TAMs (HLA‐DRA, CD68, CD3D, CD3E); and (macroC5) CXCL10 TAMs (HLA‐DRA, CD68, CD163, CXCL10). Two subpopulations were characterised as DCs: (C5) CLEC9A DCs (CLEC9A, XCR1) and (C7) activated DCs (CCR7 and LAMP3).

To better understand what distinguished these TAMs, we further characterised their six populations. We found that macrophages were enriched in primary chordomas, and the results were confirmed by IHC (Figure S12) with the macrophage markers CD68 and CD163. However, from the macrophage cluster, we inferred that there was great heterogeneity between the macrophages. We evaluated the M1 and M2 scores of TAMs, as they are frequently categorised into M1 and M2 types.^34^ Compared with macroC2, macroC1 had a higher M2 score, which revealed that macroC1 was more similar to M2 macrophages (Figure S13). Our results (Figure 3B) are consistent with the high expression of specific markers, such as CD68, CD163 and MRC1,^29^, ^35^ which are indicative of M2‐TAMs being the primary subtype of TAMs. We found that macorC1 and macroC2 both had these markers, and the macroC2 cluster had higher expression of S100A8, S100A9 and S100A12 than macroC1 (Figure 3B). The calcium‐binding proteins encoded by the above genes^36^ have been associated with inflammatory responses in vivo when released by activated mononuclear cells. GSEA revealed that interforn‐α(IFN‐α), IFN‐γ, interleukin2(IL2)/signal transducer and activator of transcriptiondao 5 (STAT5), IL6/Janus kinase (JAK)/STAT3, the IL‐17 signalling pathway, antigen processing and presentation, and inflammatory responses were among the upregulated signalling pathways in chordoma lesions, specifically in macroC1, macroC2 and macroC5 (Figure 3C). This implies that these macrophages might come from an inflammatory environment in chordoma due to the impact of IFN‐α and IFN‐γ stimulation. Of specific concern, macroC1 exhibited increased levels of the transforming growth factor‐β (TGF‐β) and Hedgehog signalling pathways (Figure 3C), which are linked to M2 polarisation. Macrophages are often linked to an immunosuppressive and tumour‐promoting phenotype, which is commonly referred to as M2 polarisation. Notably, macroC1 and macroC2 secreted VEGFA, which promotes tumour growth^35^ (Table S5). The expression of inflammatory chemokines such as CCL2, CCL4, CCL3, CXCL2, CXCL3 and CXCL3 (Table S5) characterised macroC1. Chemokines have the ability to lure different types of immune cells, such as NK cells, T cells and immature DCs, towards the microenvironment of the tumour. macroC1 was enriched in recurrent chordoma, and macroC2 was enriched in primary chordoma (Figure 3D), revealing the changes and status of macrophages in chordoma. Furthermore, RNA velocity analysis indicated a transition direction from macroC2 to macroC1, suggesting a trend of differentiation of TAMs towards the M2 macrophage phenotype in chordoma (Figure 3E). This transition may contribute to the immunosuppressive and protumoural characteristics associated with M2‐TAMs in chordoma.

The macroC5 cluster, characterised by the overexpression of proinflammatory factors such as CXCL10, would have a significant impact on drawing T cells, NK cells and DCs to the tumour microenvironment.^37^ Consistent with this finding, GSEA revealed that Macro_CXCL10 cells upregulated the inflammatory pathway (Figure 3C). This cluster was enriched in the primary chordomas (Figure 3D). Then, we focused on macroC0 and found that macroC0 was enriched in recurrent chordomas (Figure 3D). Based on the RNA velocity, macroC0 cells could differentiate into macroC5, macroC1 and macroC2 cells (Figure 3E). The M2 score of macroC0 was lower than that of macroC5, macroC1 and macroC2. There was no distinct boundary observed between M1 and M2 macrophages, as indicated by the M1 and M2 scores. Suppressing the M2‐like function of TAMs may be more effective in inhibiting the recurrence of chordomas than promoting M1‐like activity, as surgery induces the expansion of opposite‐M1 macrophages and reprograms TAMs into an M2 phenotype based on the findings.

macroC0 and macroC3 had high expression of CD24, a protein important for tumour radioresistance. Partially restoring the phagocytic ability of macrophages towards tumour cells could be achieved by controlling the breakdown of CD24 protein.^38^ These two clusters, especially macroC3, also had high expression of KRT19, which may be the reason for their radioresistance.^39^ Interestingly, macroC4 had high expression of CD3D and CD3E, which can activate TCR signalling.^40^ We found that the number of TCR clonotypes of macroC4 was reduced in recurrent chordomas (Figures 3E and S14). Furthermore, neutrophils exhibiting S100A8 and S100A9 were identified (Figure 1C). More neutrophils infiltrated the recurrent lesions than the primary lesions (Figure 1E), but the clinical significance of this difference remains to be further addressed.

### 3.4 DCs with high antigen presentation scores were enriched in the primary chordoma

The presence of two distinct subclusters of DCs in chordoma lesions, namely, CLEC9A DCs and activated DCs (Figure 3B), underscores their crucial role as major contributors to the immune response against cancerous growth. CLEC9A DCs have potent antigen‐presenting capabilities and can activate CD8 T cells, which are important for facilitating an effective antitumour immune response.^41^ Another subset of DCs identified in chordoma lesions is LAMP3+ DCs, also known as mature immunoregulatory DCs (mregDCs).^42^ The DCs expressed indicators linked to development (LAMP3), movement and spread (CCR7 and FSCN1), and showed decreased expression of Toll‐like receptors (TLRs).^42^, ^43^ Through IL‐4 stimulation,^42^ they have been involved in interactions with Treg cells that infiltrate tumours and in the suppression of tumour immunity mediated by CD8 T cell. According to trajectory analysis, mregDCs might come from cDC1s, which supports the idea that mregDCs are formed from CLEC9A DCs after absorbing tumour antigens (Figures 3G and S15).^42^, ^44^ Interestingly, after surgery, the fractions of CLEC9A DCs and mregDCs were decreased in recurrent patients (Figure 3D), indicating a decrease in the abundance of these activated DC populations. Furthermore, the antigen‐displaying pattern^23^ of DCs exhibited a notable decrease in patients with recurrence (Figure 3H), indicating a general deactivation of DCs following the surgical procedure. The implication of this discovery is that in recurrent chordoma patients, DCs, which have a vital function in activating CD8 and CD4 T cells in the TME, either become deactivated or are drawn away from the tumour location.

### 3.5 Reduced antigen presentation‐related apCAFs and elevated EMT‐related eCAFs in recurrent chordoma

**FIGURE 4:**
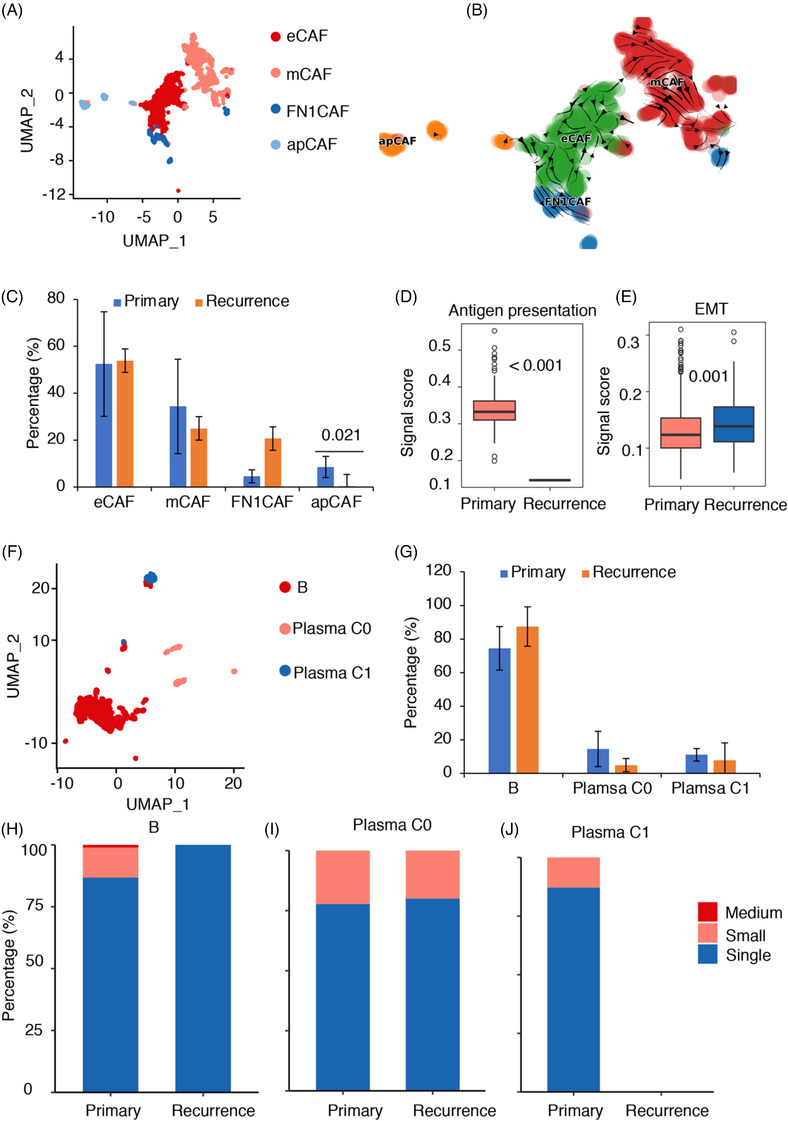
Cancer‐associated fibroblast (CAF) and B‐cell annotation and functional characterisation. (A) Uniform manifold approximation and projection (UMAP) plot of CAFs. (B) UMAP plot showing the RNA velocity of CAFs. (C) Distribution of different cell types, ranked by the median frequency value. (D) Boxplots comparing the average antigen presentation signature scores between antigen‐presenting cancer‐associated fibroblasts (apCAFs) in primary chordoma samples and recurrent chordoma samples. (E) Boxplots comparing the epithelial–mesenchymal transition (EMT) scores between EMT‐like cancer‐associated fibroblasts (eCAFs) in primary chordoma samples and recurrent chordoma samples. The one‐sided *t*‐test was applied. (F) UMAP plot of B cells. (G) Distribution of different cell types, ranked by the median frequency value. (H–J) Distribution of clone type in B cells (H), plasma C0 cells (I) and plasma C1 cells (J).

CAFs are an essential element of the tumour microenvironment (TME) and have a substantial impact on the advancement, expansion and spread of tumours.^45^ CAFs had higher expression in primary chordomas than in recurrent chordomas (Figure 1E). We found that FAP (a fibroblast marker) has higher expression in recurrent chordoma, and this result was confirmed by IHC experiments (Figures S16 and S17).^30^ Using specific markers, a total of 1796 fibroblasts were identified, which were classified into four distinct subclusters by U‐map analysis (Figures 4A and S18 and Table S6). Confirming their identity as fibroblasts, all four subclusters demonstrated elevated expression levels of well‐known fibroblast markers including DCN, LUM and COL1A2.^8^, ^29^

Subcluster 1, consisting of 758 fibroblasts, displayed upregulation of genes such as KRT19, LGALS3 and KRT8 (Table S6), which led to its designation as eCAFs.^8^, ^46^ Notably, this subcluster also exhibited high expression of CD24, potentially contributing to the protection of eCAFs from cytotoxic cell attacks. Subcluster 2, comprising 685 cells, exhibited extracellular matrix (ECM) characteristics, such as collagen proteins (COL1A1, COL1A1, COL3A1, COL4A1, COL14A1), DCN and LUM.^8^, ^29^ GO analysis associated this subtype with ECM organisation, wound healing, collagen fibril organisation and smooth muscle cell proliferation (Table S7), leading to its designation as matrix cancer‐associated fibroblasts (mCAFs). With 154 fibroblasts, subcluster 3 exhibited relatively low expression of KRT19, LGALS3 and KRT8 but displayed the highest expression of FN1. Consequently, these cells were labelled FN1 cancer‐associated fibroblasts (FN1CAFs). Finally, subcluster 4 had 127 fibroblast cells and showed significant upregulation of genes such as CD74, CCL5, PTPRC, CXCR4 and HLA‐DRB1 (Table S6).^8^, ^47^ GO analysis confirmed the involvement of cell chemotaxis, the antigen receptor signalling, and the TCR pathway. This subcluster was named apCAFs (Table S7).

By employing RNA velocity analysis, we observed a transition direction from apCAFs towards other CAF subtypes (Figure 4B). This finding suggests that apCAFs have the potential to differentiate into FN1CAFs and mCAFs, which may be associated with the formation of primary and recurrent chordomas, respectively. Intriguingly, we discovered a significant enrichment of apCAFs in recurrent chordomas (Figure 4C). When we compared the antigen presentation capability of apCAFs between primary and recurrent chordomas, we found that the antigen presentation ability was significantly enhanced in primary chordomas (Figure 4D). As for eCAFs, although their percentage decreased in recurrent chordomas, the EMT score rose (Figure 4E).

To clarify the biological role of CAFs, an analysis of cell‒cell communication was carried out utilising CellChat.^25^ Remarkably, FN1CAFs showed elevated levels of ligands linked to EMT, including collagens (COL1A1, COL1A2, COL2A1, COL6A2), FN1 and LAMININ (LAMC1, LAMB1, LAMB2, LAMB1). These ligands engaged with receptors expressed by various cell types, leading to the initiation of specific cell‒cell interactions (Figure S19A,B). Notably, we observed the absence of these signalling pathways in apCAFs from recurrent chordomas (Figure S20). Integrins (ITGA1, ITGA2, ITGA3, ITGA4, ITGA5, ITGA8, ITGAV, ITGAX, ITGAM, ITGB1 and ITGB2) expressed in tumours have the ability to identify various ligands such as collagens, FN1 and LAMININ. This leads to subsequent communication between tumours and CAFs. The signalling of integrin has a significant impact on cancer cells, influencing their growth, movement and ability to survive.^48^ Integrins (ITGA1, ITGA2, ITGA3, ITGA4, ITGA5, ITGA8, ITGAV, ITGAX, ITGAM, ITGB1 and ITGB2), which are encoded by specific genes, are receptors expressed in tumours. These integrins have the ability to identify various ligands, such as collagens, FN1 and LAMININ. As a result, they promote communication between tumours and CAFs through downstream crosstalk. The behaviours of tumour cells, such as their proliferation, migration and survival, are significantly influenced by integrin signalling.^48^ ITGA3 has been linked to enhancing the movement of endothelial cells and the formation of new blood vessels in the early phases of neovascularisation.^49^ Hence, communication between CAFs and tumours through multiple signalling pathways, including integrin‐related pathways, may contribute to the promotion of EMT in chordomas.

### 3.6 Reduced number of plasma cells and decreased BCR clonotypes in recurrent chordoma

A total of 2055 B‐cell populations underwent unsupervised clustering, resulting in the identification of three distinct subclusters characterised by their unique signature genes (Figures 4F and S21 and Table S8). One cluster was identified as B cells (CD19, CD79A, CD79B and MS4A1); two clusters were identified as plasma cells: plasma C0 (CD19, CD27, MS4A1, IGHG3, IGHA1, DERL1, FKBP11, MZB1 and JCHAIN) and plasma C1 (CD19, MS4A1, MZB1, IGHA1, DERL1 and JCHAIN). We found that B cells were more abundant in recurrent chordomas, while the other two plasma cell types were more abundant in primary chordomas (Figure 4G).

To examine the clonal connections among tissues, we employed scBCR‐seq data on V(D)J in combination with scRNA‐seq data. A total of 2203 cells were found to have heavy and corresponding light chains that could be aligned with scRNA‐seq data. Importantly, the medium‐sized clonotype groups contained the majority of B cells, while unexpanded cells, represented by small and single clonotypes, included mainly plasma cells (Figure S22). We observed that B cells had more clonotypes in the primary chordomas (Figure 4H). Of note, the medium clonotypes in the B cells were only spread across the primary chordomas (Figure 4H). We noticed that plasma cells in the primary chordomas had more clonotypes (Figure 4G,I). There were fewer plasma cells in the recurrent chordomas (Figure 4G). The findings underscore the possible importance of B cells in controlling the advancement of chordoma, underscoring the necessity for additional research in this domain.

### 3.7 Elevated EMT‐related FN1 expression in recurrent chordoma

**FIGURE 5:**
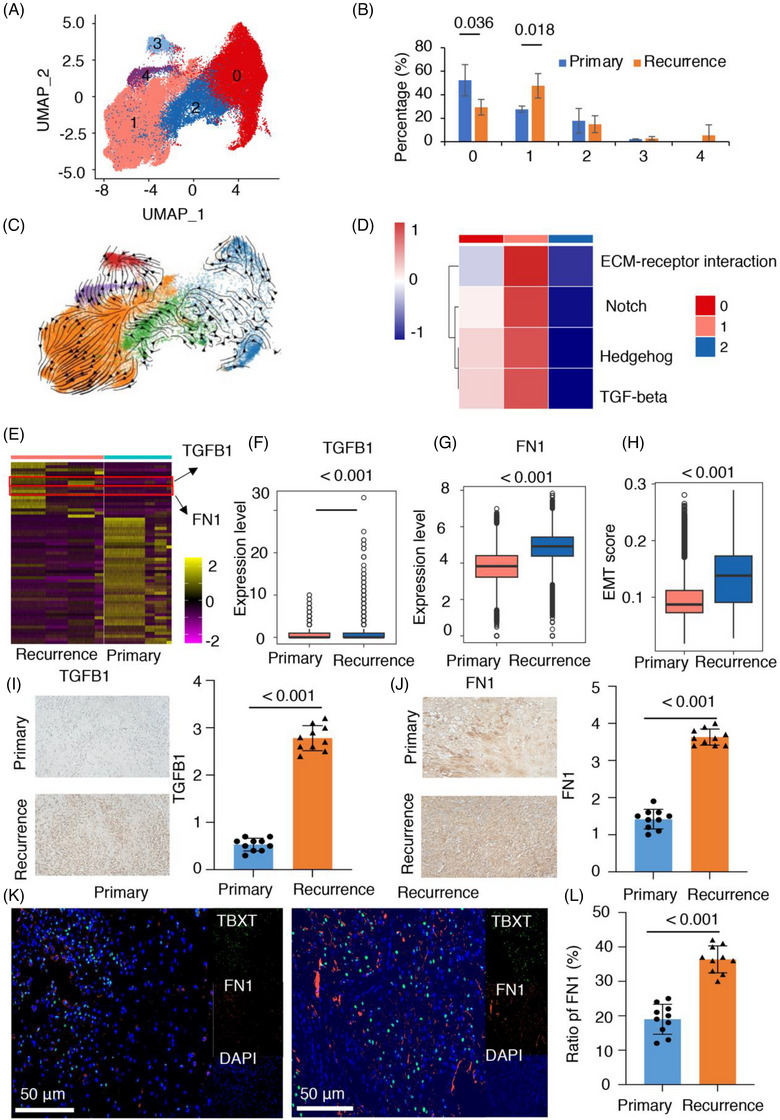
Elevated epithelial–mesenchymal transition (EMT)‐related fibronectin 1 (FN1) expression in recurrent chordoma. (A) The major cell populations of tumour cells were visualised using uniform manifold approximation and projection (UMAP), with differently coloured dots representing different cell types. (B) Distribution of different cell types, ranked by the median frequency value. (C) UMAP visualisation showing the RNA velocity of tumour cells. (D) Gene set enrichment analysis (GSEA) results of tumour cells. (E) differentially expressed genes (DEGs) between primary and recurrent chordomas in tumour C1 cells. (F and G) The expression of FN1 and TGFB1 between primary and recurrent chordomas. (H) Distribution of EMT scores between primary and recurrent chordomas. (I) Immunohistochemistry (IHC) for TGFB1 in primary and recurrent chordoma samples (*n* = 10). (J) IHC for FN1 in the primary and recurrent chordoma samples (*n* = 10). (K and L) Representative multiplex IHC (mIHC) images of FN1, TBXT and DAPI in primary and recurrent chordomas (*n* = 10).

Next, we focused on 71 215 malignant cells, including 41 142 malignant cells from primary chordomas and 30 073 malignant cells from recurrent chordomas, and identified five subtypes of malignant cells (Figures 5A and S23) with differentially expressed genes (DEGs) (Table S9). In total, five populations were designated tumour clusters, which had various features: (TumourC0) CD74 and APOD; (TumourC1) IF16, JUN and HSPA1A; (TumourC2) S100B and SCRG1; (TumourC3) NEAT1 and MALAT1; and (TumourC4) WFDC2 and RNASE1 (HLA‐DRA, CD68, CD3D, CD3E). Furthermore, all clusters exhibited high expression of the mesenchymal cell signature (VIM), while none of the clusters expressed any of several epithelial markers (Figure S24). In addition to cluster 4, which was present in all eight patients, the other clusters included cells from all patients, demonstrating significant tumour heterogeneity within and between primary and recurrent chordomas (refer to Figures S25 and S26). The results indicate that the diversity of cancer cells plays a role in the differences observed in the TME between primary and recurrent chordomas.

Clusters 1, 3, and 4 were enriched in recurrent chordomas, and the others were enriched in primary chordomas (Figure 5B). Subsequently, we performed RNA velocity analysis on the five clusters and observed that cluster 1 exhibited a more primitive or progenitor stage (Figure 5C). Notably, TumourC1 showed a significant enrichment in recurrent chordomas (Figure 5B). Additional examination through GSEA indicated that TumourC1 cells were linked to signalling pathways such as TGF‐β, Notch, ECM–receptor interaction and Hedgehog, which have been connected to the advancement of tumours and the ability to resist cancer treatments (Figure 5D).^7^, ^8^, ^50^, ^51^ Given these findings, we focused on investigating the role of TumourC1 in chordoma recurrence. In the analysis of TumourC1 cells, we found DEGs between primary and recurrent chordomas. Notably, we observed an upregulation of genes associated with EMT, including TGFB1 and FN1, in recurrent chordomas (Figure 5E–G, and detailed in Table S10). The recurrent chordomas exhibited a higher EMT score than primary chordomas (Figure 5H). These findings suggest that the activation of EMT‐related pathways may contribute to the recurrence of chordomas.

To confirm the expression levels of TGFB1 and FN1 in chordoma and their correlation with recurrence, we performed IHC on clinical samples including 10 primary chordomas and 10 recurrent chordomas, which revealed significantly higher expression levels of TGFB1 and FN1 in recurrent chordoma than in primary tumours (Figure 5I,J), indicating a potential association between EMT and chordoma recurrence. Furthermore, we conducted immunofluorescence staining of FN1 and the chordoma marker TBXT. The examination uncovered a separate group of chordoma cells that displayed robust FN1 expression concentrated at the forefront of the tumour (as shown in Figure 5K,L). This discovery implies that FN1 might have a part in the invasive nature of chordoma cells and their movement within the microenvironment of the tumour.

### 3.8 Single‐cell CNV analysis revealed that recurrent chordoma has a higher level of FN1 copy number gain

**FIGURE 6:**
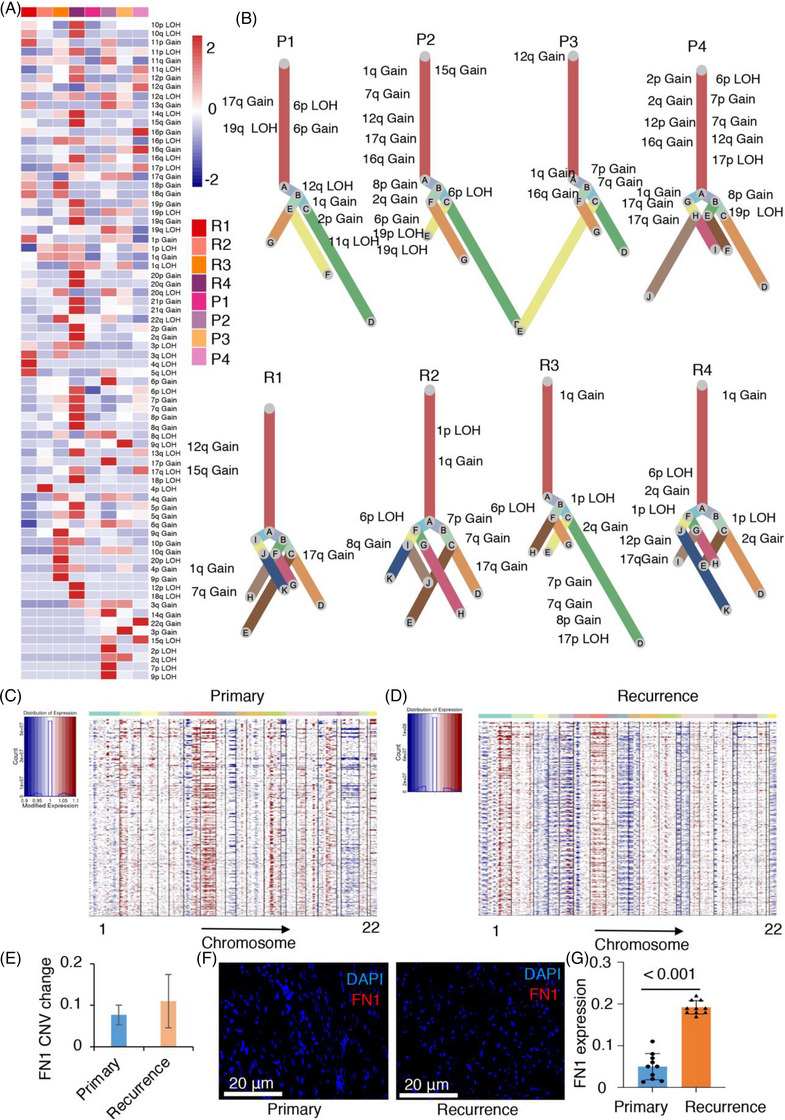
The frequency of copy number gain of fibronectin 1 (FN1) is increased in recurrent chordoma cells. (A) The inferred copy number variant (CNV) profiles of chordoma cells from eight samples were summarised using inferCNV analysis. The CNV levels were categorised by chromosome arm as gains or losses. The heatmap displays the percentage of CNV events in single cells for each individual sample. (B) Clonality trees visualising the subclonal composition of single cells, with branches representing the percentage of cells in each subclone containing corresponding CNVs. (C and D) The hierarchical heatmap further illustrates the large‐scale CNV patterns in primary and recurrent chordoma samples. (E) FN1 had a higher frequency of copy number gain in recurrent chordoma than in primary chordoma. (F and G) Fluorescence in situ hybridisation (FISH) results of FN1 in primary and recurrent chordomas (*n* = 10).

Chordoma is distinguished by notable somatic copy number changes and structural variations, with limited recurring point mutations in genes that code for proteins.^52^ To examine the clonal arrangement of chordoma cells, we employed the inferCNV algorithm to examine CNVs in individual cells obtained from each lesion.^29^, ^53^ Using the UPhyloplot2 plotting algorithm,^21^ we created clonality trees for chordoma lesions based on the combined CNV findings. The analyses uncovered the existence of numerous standard (CNV percentage > 90%) and nonstandard CNVs (CNV percentage < 90%) in subclones present within every lesion (Figure 6A,B). By investigating the clonal composition of every tumour, we discovered an unforeseen intricacy in the presence of both typical and atypical CNVs (Figures 6A,B and S27). We consistently observed common chromosomal alterations in the lesions, such as amplifications of 7q, 1q, 2q and 2p, as well as loss of heterozygosity in 19q, 19p and 6p. The results align with the genomic CNVs previously detected in comparative genomic hybridisation and whole‐genome sequencing investigations.^52^, ^54^ Notably, 1q gain was more frequently observed in recurrent chordomas than in primary chordomas (Table S11). The examination of clonality revealed the previously unrecognised intricacy of both typical and atypical CNVs in chordoma (Figure 6A,B). Canonical CNVs dominated the chromosomal landscape, but multiple subclonal canonical and noncanonical CNVs were observed among chordoma patients, indicating the presence of subclonal cellular populations in tumour cell evolution. Notably, a higher incidence of typical CNVs was noted in initial tumours compared to recurrent chordoma specimens (Figure 6A,B). Additional research is necessary to validate the importance of these CNVs. There were stark differences between primary and recurrent chordomas (Figure 6C,D). FN1 had more CNVs in the recurrent lesions than in the primary lesions, which was confirmed by FISH experiments on the 10 primary and 10 recurrent chordomas (Figure 6E–G and Table S12). The results emphasise the complex clonal organisation and genetic diversity of chordoma, emphasising the significance of comprehending the impact of CNVs on the advancement and reappearance of tumours.

### 3.9 FN1 is readily secreted in CD4 T cells, CD8 T cells, macrophages and fibroblasts of recurrent chordoma

The current evidence supports the presence of differences in the composition and phenotype of the TME between primary and recurrent chordomas.^55^ The analysis of cell‒cell communication provided support for our theory about different subcellular interaction relationships in the TME of primary and recurrent chordomas. Specifically, recurrent chordomas exhibited a higher number of cell interaction events and stronger interaction, despite the smaller cell population, than primary chordomas (Figure S28). Moreover, the examination of the strengths of interactions coming in and going out emphasised the notable contribution of cancerous cells in the TME of recurrent chordomas in contrast to primary chordomas (Figure S29A,B).

**FIGURE 7:**
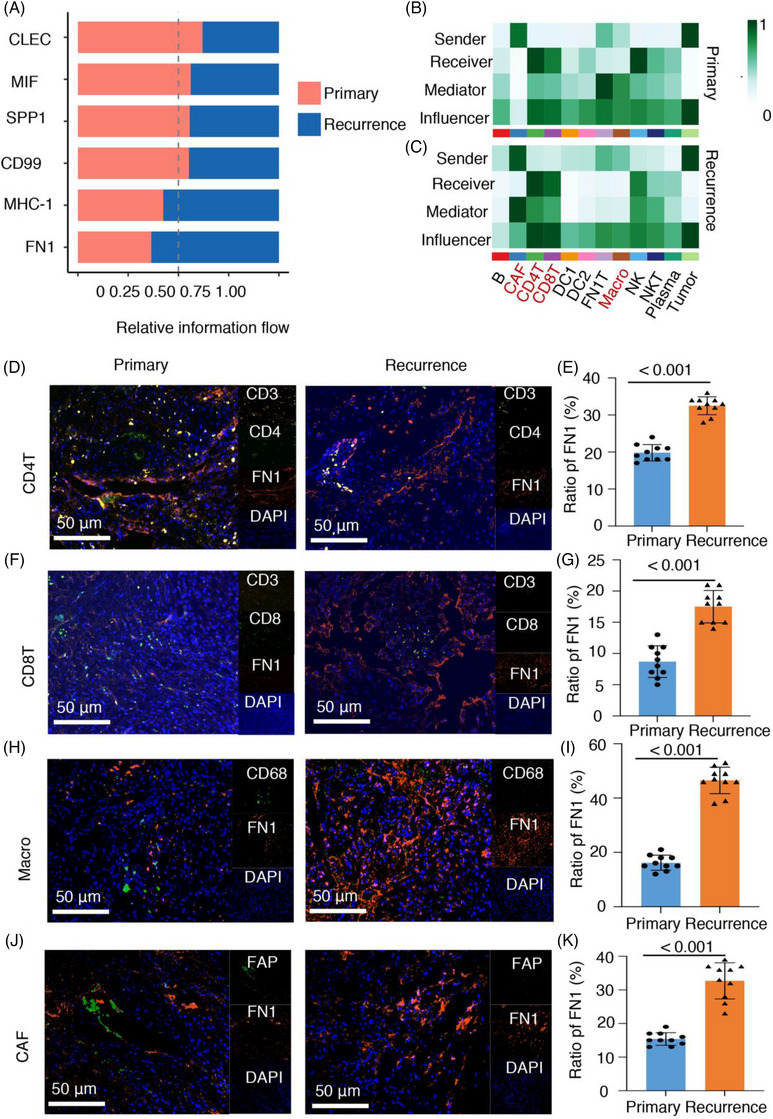
Fibronectin 1 (FN1) was enriched in recurrent chordomas among CD4 T cells, CD8 T cells, macrophages and cancer‐associated fibroblasts (CAFs). (A) FN1 signalling information flow fraction by sample type. (B and C) Heatmap of the relative importance of each cell group based on the four computed network centrality measures of FN1 signalling in primary (C) and recurrent chordomas (C). (D and E) Representative images of CD3, CD4, FN1 and DAPI staining in primary and recurrent chordomas using multiplex immunohistochemistry (mIHC) (*n* = 10). (F and G) Representative images of CD3, CD8, FN1 and DAPI staining in primary and recurrent chordomas using mIHC (*n* = 10). (H and I) Representative images of CD68, FN1 and DAPI staining in primary and recurrent chordomas using mIHC (*n* = 10). (J and K) Representative images of FAP, FN1 and DAPI staining in primary and recurrent chordomas using mIHC (*n* = 10).

Afterward, we conducted a targeted examination of cancerous cells and noticed a heightened level of communication signals in recurrent chordoma when compared to the primary chordoma control group. Notably, the FN1 pathway showed significant upregulation in recurrent chordoma versus primary chordoma, highlighting its potential role in disease progression (Figure 7A). Prior research has emphasised the vital function of FN1 as a pivotal regulator implicated in diverse facets of cancer progression, encompassing cellular evolution, proliferation, viability, movement and blood vessel formation, in numerous forms of human cancer.^56^ Our analysis revealed that the interaction between FN1 and CD44, as well as the interaction between FN1 and ITGA4/ITGB1, plays prominent roles in the communication network of both primary and recurrent chordomas (Figure S30A,B). Interestingly, in recurrent chordomas, interactions involving FN1 and ITGAV/ITGB1, as well as FN1 and ITGA3/ITGB1, emerged as significant contributors to this communication network. Then, we found that more cells in the TME could be the ‘sender’ in cell–cell communications in recurrent chordomas than in primary chordomas. There were clear clusters of TILs, macrophages and fibroblasts. The recurrent chordomas exhibited a high abundance of CD4 T cells, CD8 T cells, macrophages and CAFs, as shown in Figure 7B,C. These data indicate important changes in the TME of chordomas after recurrence. The expression of FN1 in CD4 T cells, CD8 T cells, macrophages and CAFs was calculated, and FN1 was highly concentrated in recurrent chordomas (Figure S31A–D).

To validate the results, we performed mIHC on 10 primary chordomas and 10 recurrent chordomas. Initially, we investigated the impact of FN1 on CD4 T cells and CD8 T cells by analysing the presence of CD3D, CD4, CD8 and FN1 in both primary and recurrent chordoma specimens. The recurrent chordoma samples expressed FN1 at a higher level than the primary samples, suggesting increased FN1 presence in T cells (Figure 7D–G). Likewise, upregulation of FN1 was observed in macrophages (Figure 7H,I) and fibroblasts (Figure 7J,K) from recurrent chordoma samples in comparison to primary samples. The results suggest that FN1 may play a role in controlling immune cells and stromal components in the microenvironment of recurrent chordoma. The combined results suggest that FN1 secretion, specifically in CD4 T cells, CD8 T cells, macrophages and fibroblasts, may be increased in the TME of recurrent chordomas. Consequently, targeting FN1 shows promise as a strategy to inhibit chordoma recurrence.

### 3.10 FN1 is a potential target in vivo and in vitro

**FIGURE 8:**
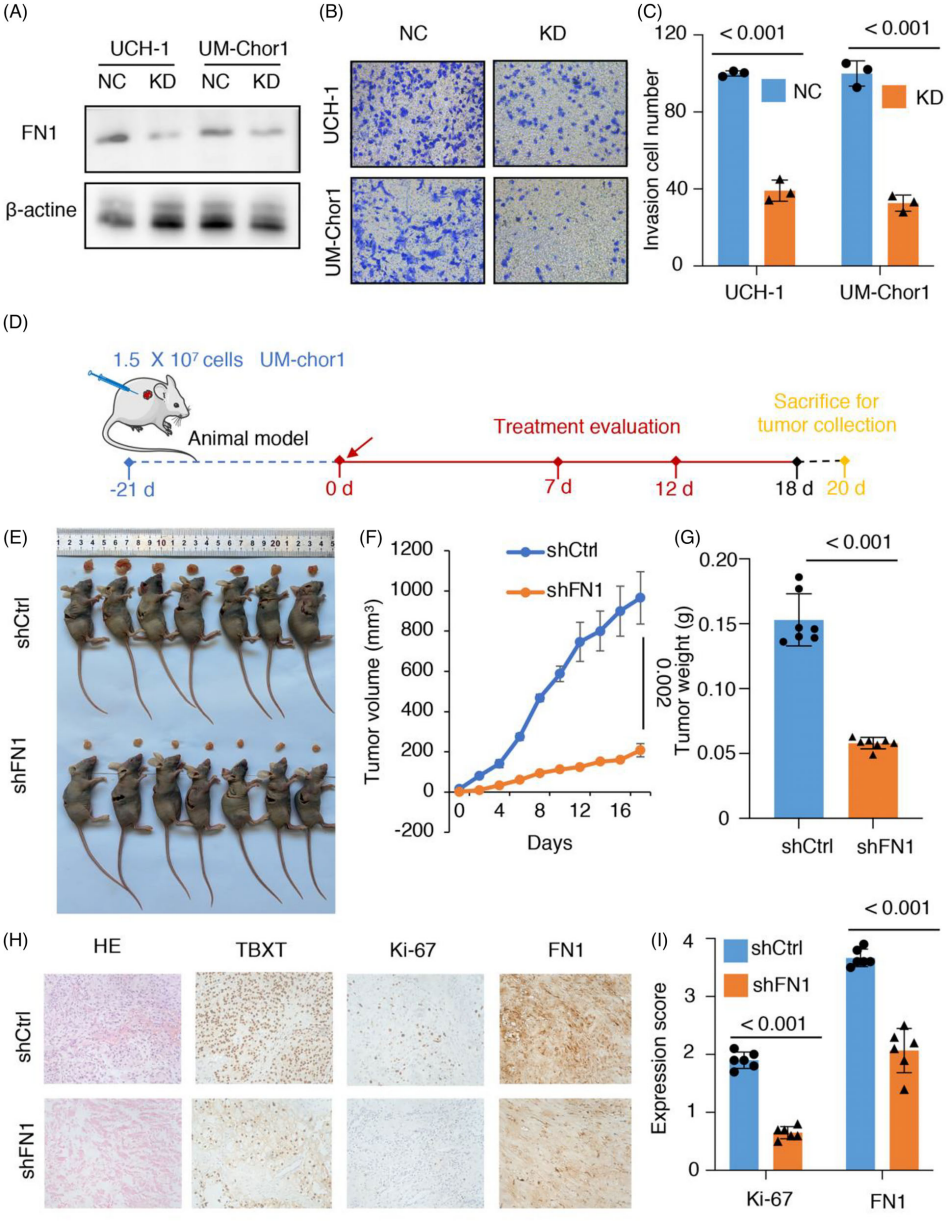
Fibronectin 1 (FN1) is a potential target in vitro and in vivo. (A) Fractionation analysis of FN1 between the shCtrl and shFN1 groups by Western blot. (B and C) The Transwell results between the shCtrl and shFN1 groups of U‐CH1 and UM‐chor1 cells (*n* = 3). (D) Schematic illustration of tumour inoculation and treatment protocol for UM‐chor1 xenograft tumour‐bearing mice. (E) Representative images of harvested UM‐chor1 tumours and of mice after treatment with shCtrl and shFN1 (*n* = 7). (F) Volume of harvested UM‐chor1 tumours in the shCtrl and shFN1 groups (*n* = 7). (G) Weight of harvested UM‐chor1 tumours in the shCtrl and shFN1 groups (*n* = 7). (H and I) Immunohistochemistry (IHC) for TBXT, Ki‐67 and FN1 between the primary and recurrent samples (*n* = 6).

To validate the functional importance of FN1 and evaluate the possibility of targeting FN1, we performed experiments to suppress the expression of FN1 in UM‐chor1 cells and U‐CH1 cells. Western blot analysis revealed high levels of FN1 protein expression in both cell lines (Figure 8A). Upon assessing the impact of FN1 depletion on cellular behaviour, it was noted that the shFN1 group exhibited a considerably reduced migration ability compared to the shCtrl group, as indicated by the fold change (*p* < .001). This suggests that the suppression of FN1 expression hindered the ability of cells to migrate (Figure 8B,C). To further explore the function of FN1 in the regulation of chordoma, we employed a mouse xenograft model (Figure 8D). Through animal experiments, the size and dimensions of xenograft tumours were assessed and computed. The findings indicated a considerably reduced rate of growth and a smaller ultimate size of the tumour in the shFN1 group compared to the shCtrl group (*p* < .01) (Figures 8E,F and S32). Furthermore, IHC analysis of the tumour tissues demonstrated decreased levels of Ki67 and FN1 expression in the shFN1 group compared to the shCtrl group (*p* < .05) (Figure 8G). The results suggest that suppressing FN1 can hinder the growth of tumours in mice, providing further evidence of FN1's involvement in promoting chordoma development. Collectively, the findings from the experiments suggest that the reduction in FN1 expression suppresses the proliferation of tumours cells and hinders malignant behaviours, such as cell movement, in vivo and in vitro.

## 4 DISCUSSION

Despite being an uncommon form of cancer, over half of chordoma patients relapse,^4^ and approximately 30%−40% of patients will experience metastasis to other locations after recurrence,^57^ which often leads to death.^52^, ^58^ Through this investigation, we performed single‐cell RNA sequencing on numerous individual cells obtained from primary and recurrent chordomas, leading to a thorough comprehension of the operational traits and cellular interactions within the TME. In both primary and recurrent chordoma samples, we examined and studied different types of cells, such as tumour cells, fibroblasts, neutrophils, macrophages, DCs, B cells and T cells. Through our comprehensive analysis, we discovered a noteworthy transformation of the TME in primary and recurrent chordomas, providing insights into the fundamental causes of chordoma recurrence. The implications of these findings are significant for the advancement of clinical approaches focused on preventing and treating the recurrence of chordoma. This is particularly important due to the observed variability in tumour characteristics within and among tumours. Furthermore, the identification of distinctive features between primary and recurrent chordomas provides valuable insights into the mechanisms driving different clinical outcomes in chordoma patients.

Immune cells, which are a crucial part of the TME, frequently demonstrate suppressive effects on the immune system.^30^ The TME consists of a wide range of cellular components that enable interactions among cancerous cells, immune cells and stromal cells.^59^ In recurrent chordoma, our research uncovered a significant decline in the infiltration of T cells and an increase in T‐cell exhaustion. Additionally, a prior investigation indicated that increased T‐cell infiltration correlated with a more favourable prognosis.^60^ Tumour‐infiltrating CD8 T cells frequently present exhausted phenotypes in different types of tumours,^61^ such as cutaneous squamous cell carcinoma,^47^ melanoma^62^ and breast cancer.^63^ CD8 T cells in a state of exhaustion exhibit elevated levels of immune checkpoint receptors including PD‐1, CTLA‐4, TIM‐3 and LAG‐3.^64^ Malfunction of these cells^65^ is correlated with postoperative survival and the likelihood of recurrence. We also observed this phenomenon. In addition, we found that there was a transition from CD8_GZMK T cell to CD8_ZNF683 T cell, and CD8_ZNF683 T cell had a higher exhaustion score and lower effector score. On the other hand, NKT cells could transform into NK cells, but NKT cells had a higher exhaustion score and lower effector score. We infer that promoting NK and CD8_GZMK T‐cell function or inhibiting the transformation of CD8GZMK T cell to CD8_ZNF683 T cell and promoting the transformation of NKT cells to NK cells may be promising strategies for preventing chordoma recurrence. ‘Cold tumours’, which exhibit a scarcity of effector T cells, are resistant to immunotherapy.^66^ Therefore, the limited presence of effector T cells in recurrent chordoma suggests that immunotherapy may not be an effective treatment modality for this condition. We also found higher TCR modalities in primary chordoma. In summary, our results suggest that primary chordoma displays attributes of a ‘hot’ tumour, with an increased presence of infiltrating CD4 T and CD8 T cells, whereas recurrent chordoma shows characteristics of a ‘cold’ tumour, along with EMT characteristics.

M2‐TAMs, known for their elevated MRC1 expression, play a vital role in the advancement of tumours, encompassing tumour expansion, formation of new blood vessels, infiltration and metastasis.^67^ Our findings revealed that TAMs predominantly displayed the M2 subtype, which contributes to angiogenesis promotion and immunosuppressive effects within the tumour microenvironment.^68^, ^69^ We found that there were changes in the transition from non‐M2 macrophages to M2 macrophages. These findings indicate the potential involvement of M2 macrophages in chordoma progression, highlighting the need for further research to elucidate their specific roles and underlying mechanisms. In conclusion, we infer that suppressing M2‐TAMs in chordomas may be an effective strategy. Interestingly, macroC4 had a high expression of CD3, and in recurrent chordoma, the TCR clone types were less common. The characteristics of TCR (+) TAMs are unclear, but there are some studies about this kind of TAM inflammatory and infectious disease.^69^, ^70^ Whether TCR (+) TAMs are associated with inflammatory and infection‐related changes in chordoma is unknown, so more work is needed. These findings indicate that the myeloid cells in chordomas have a protumour status and reduced antitumour ability. We also found a transition in DCs, and their antigen presentation ability was reduced in recurrent chordomas. The presence of DCs in chordoma lesions, their capacity for antigen presentation, and their potential to activate T cells make them promising targets for immunotherapy strategies. By understanding the dynamics and functional states of DC populations in chordoma, researchers can explore ways to reactivate and enhance their antigen‐presenting abilities, thereby promoting a more robust antitumour immune response.

CAFs in the TME play a dual role in the advancement of tumours. They possess the capacity to both impede and facilitate tumour growth, and have been linked to the initiation of EMT in different cancer forms.^71^, ^72^, ^73^ Here, we found that eCAFs had a higher EMT score in recurrent chordoma. apCAFs were enriched in primary chordoma, and their antigen presentation ability was better than that of recurrent chordoma. Additionally, our findings revealed that FN1CAFs were present at higher levels in recurring chordoma. These FN1CAFs were capable of engaging with tumours by expressing ligands associated with EMT, including collagens, FN1 and LAMININ, primarily through collagens, FN1 and LAMININ interactions. Hence, the interaction between tumours and FN1CAFs may have a significant impact on facilitating the EMT process of tumour cells in recurrent chordoma. We found that there were fewer BCR clonotype of B cells in recurrent chordomas, although B cells were enriched in recurrent chordomas. There were also fewer BCR clonotypes of plasma cells in recurrent chordomas. Overall, recurrence introduced some change in the activation of plasma cells.

Others have proposed the concept of an EMT phenomenon in chordomas.^7^ We found that FN1 was an important factor promoting EMT changes in recurrent chordoma cells. Although there have been no studies on FN1 in chordoma, FN1 can promote the malignant progression of bone tumours, such as osteosarcoma.^74^, ^75^ In addition, the well‐known EMT marker TGFB1 has been observed in recurrent chordomas.^7^, ^76^ To verify the increased expression of FN1 and TGFB1 in recurrent chordoma, we performed IHC analysis in a separate clinical group, which further confirmed their expression levels in recurrent chordoma. This study aimed to clarify the precise function and underlying molecular mechanism of FN1 in chordoma. In vitro, the migration of chordoma cells was significantly hindered by the knockdown of FN1. Consistent with this finding, downregulation of FN1 led to a suppression of chordoma growth in an in vivo model, supporting its potential as a therapeutic target for chordoma treatment. Through CNV analysis, we found that FN1 had a higher frequency of copy number gain in recurrent chordomas, as verified by FISH.

In addition, our findings highlight that FN1 was upregulated in recurrent chordomas compared with primary chordomas, and tumour cells, CD8 T cells, CD4 T cells and macrophages also secreted FN1 to promote the malignant progression of chordomas, demonstrating its ability to drive robust intercellular interactions within the TME of recurrent chordomas. Notably, the presence of FN1 appears to profoundly influence various malignant biological factors, including the ECM and acquisition of epithelial‐like characteristics, in tumour cells.^56^, ^77^ The downregulation of FN1 may further enhance the inhibitory effects on chordoma, making it a promising candidate for targeted treatment. Moreover, our study reveals a distinct transformation pattern of FN1 expression from primary chordoma to recurrent chordoma, indicating its significance as a novel therapeutic target in chordoma. In summary, these results highlight the essential role of FN1 in the progression of tumours and the suppression of the immune system in chordomas.

To summarise, our research provides a thorough examination of the transcriptomic and immune characteristics of primary and recurrent chordomas at the single‐cell scale, emphasising the importance of FN1 as a pivotal protein in the recurrence of chordomas. These findings contribute valuable insights into the molecular mechanisms underlying chordoma recurrence and provide potential therapeutic avenues for targeting FN1 in the management of chordoma patients.

## CONFLICT OF INTEREST STATEMENT

The authors declare they have no conflicts of interest.

## Supporting information

Supporting Information

Supporting Information

## Data Availability

The raw sequencing data that support the findings of this study were deposited in the Genome Sequence Archive of Beijing Institute of Genomics, Chinese Academy of Sciences: HRA004916). All the other data supporting the findings of this study are available within the article and its supplementary information files, without any restrictions or from the corresponding author, upon reasonable request. Source data are provided with this paper.
