# Supplementary material for: Unravelling the role of immune cells and FN1 in the recurrence and therapeutic process of skull base chordoma
Source: Clin Transl Med. 2023 Oct 2;13(10):e1429. doi: 10.1002/ctm2.1429 (PMC10545891; doi:10.1002/ctm2.1429)
Supplement: Supplementary file 1 — Supporting Information [file CTM2-13-e1429-s001.docx]

**Unraveling the role of immune-cells and FN1 in the recurrence and therapeutic process of skull base chordoma**

Xulei Huo^1^, Sihan Ma^1^, Bohan Yao^1^, Sipeng Zhu^1^, Lairong Song^1^, Can Wang^2^, Da Li^1^, Junpeng Ma^1^, Liang Wang^1^, Zhen Wu^#1^, and Ke Wang^#1^

**Affiliation**: ^1^Department of Neurosurgery, Beijing Tiantan Hospital, Capital Medical University, Beijing, China.

^2^ Department of Neuro-oncology，Cancer Center，Beijing Tiantan Hospital，Capital Medical University.

^#^The corresponding authors

**Running title**: A single-cell analysis of primary and recurrent chordoma

**Keywords**

chordoma; Single Cell; FN1; Immunosuppressive

**Financial support**: This work was supported by the National Natural Science Foundation of China (No. 62027813, and 2022YFE0112500).

**Corresponding authors**: Zhen Wu, email: [wuzhen1966@aliyun.com](mailto:wuzhen1966@aliyun.com), Tel: +86-15601125551, Department of Neurosurgery, Beijing Tiantan Hospital, Capital Medical University, Nansihuanxilu 119, Fengtai District, Beijing, 100070, China; Ke Wang, wangke0624ttyy@163.com.

**Conflict of Interest**: All authors indicated no potential conflicts of interest.

**Supplementary Figures**

**Figure S1**. The immunohistochemistry of HE, and TBXT in the eight chordoma samples.

**Figure S2**. Cell type fractions relative to the total cell count per sample.

**Figure S3.** Uniform Manifold Approximation and Projection (UMAP) of cell types among chordomas, different colored dots represent different cell types.

**Figure S4**. Dot plots showing cluster signature genes in T/NK cells. The size of dots represents the proportion of cells expressing the particular marker, and the spectrum of color indicates the mean expression levels of the markers.

**Figure S5.** Distribution of different cell types of TILs, ranking by the median value.

**Figure S6**. The immunohistochemistry of CD4, CD8, CD56, and Foxp3 in primary and recurrent chordomas (n=10).

**Figure S7**. The Monocle 2 trajectory plot showing the dynamics of CD8 T cells (A) and their pseudotime curve (B).

**Figure S8**. The Monocle 2 trajectory plot showing the dynamics of NKT and NK cells (A) and their pseudotime curve (B).

**Figure S9**. UMAP visualization of TCR clone type in T/NK cells.

**Figure S10**. Distribution of TCR clone type in TILs.

**Figure S11**. Distribution of TCR clone type in NK cells, related to 2I.

**Figure S12**. The immunohistochemistry of CD68, and CD163 in primary and recurrent chordomas (n=10).

**Figure S13**. The M0, M1, and M2 score of macrophages.

**Figure S14**. UMAP visualization of TCR clone type in TAMs.

**Figure S15**. The pseudotime curve showing the dynamics of DC cells, related to 3G.

**Figure S16**. Boxplots of average FAP expression levels for CAF in primary samples and recurrent samples. One-sided t-test was used.

**Figure S17**. The immunohistochemistry of FAP in primary and recurrent chordomas (n=10).

**Figure S18**. Dot plots showing cluster signature genes in CAFs. The size of dots represents the proportion of cells expressing the particular marker, and the spectrum of color indicates the mean expression levels of the markers.

**Figure S19**. Heatmap showing the outgoing (A) and incoming (B) communication patterns of the whole distinct cell types in the primary and recurrent chordomas.

**Figure S20**. Dot plot showing the Predictive heatmap of ligand-receptor pairs between CAFs and Tumors.

**Figure S21**. Dot plots showing cluster signature genes in B cells. The size of dots represents the proportion of cells expressing the particular marker, and the spectrum of color indicates the mean expression levels of the markers.

**Figure S22**. UMAP visualization of BCR clone type in B cells.

**Figure S23**. Uniform Manifold Approximation and Projection (UMAP) of tumor cells between primary and recurrent chordomas, different colored dots represent different cell types.

**Figure S24**. The expression level of mesenchymal cell signature and epithelial markers among different cell types.

**Figure S25**. Sample type fractions relative to the total cell count per cell type in Tumor cells.

**Figure S26**. Sample fractions relative to the total cell count per cell type in Tumor cells.

**Figure S27**. Copy-number variation analysis for 8 chordoma samples.

**Figure S28**. The number interaction and the strength in primary and recurrent chordomas among whole cells.

**Figure S29**. The number interaction and the strength in primary and recurrent chordomas among different cell types.

**Figure S30**. Relative contribution of each ligand-receptor pair to the overall communication network of FN1 signaling pathway.

**Figure S31**. Boxplots of average FN1 expression levels for CD4T, CD8T, Macrophages, and CAF in primary samples and recurrent samples. One-sided t-test was used.

**Figure S32**. Representative images of harvested UM-chor1 tumors after treatment with shCtrl and shFN1 (n=7).

**Supplementary Figures**

**Table S1.** The detailed information of chordomas

**Note:** As for the primary chordomas, the pre-radiotherapy means the patient got the radiotherapy after surgery. As for the recurrent chordoma, the pre-radiotherapy means the patient got the radiotherapy after initial surgery.

**Table S2**. Antibody Information. Antibody targets with respective conjugated heavy metals (if used for IHC), dilution factor, antibody clone, clone number, and manufacturer information.
